# Primary total knee arthroplasty using constrained condylar knee design for severe deformity and stiffness of knee secondary to post-traumatic arthritis

**DOI:** 10.1186/s13018-018-0761-x

**Published:** 2018-04-02

**Authors:** Saroj Rai, Xianzhe Liu, Xiaobo Feng, Bimal Rai, Nira Tamang, Jing Wang, Shunan Ye, Shuhua Yang

**Affiliations:** 1 Department of Orthopaedics, Union Hospital, Tongji Medical College, Huazhong University of Science and Technology, Wuhan, 430022 China; 2 Department of Radiology, Tongji Hospital, Tongji Medical College, Huazhong University of Science and Technology, Wuhan, 430030 China; 3 School of Nursing, Tongji Medical College, Huazhong University of Science and Technology, Wuhan, 430030 China

**Keywords:** Total knee arthroplasty, Constrained condylar knee, Knee Society Score, Post-traumatic arthritis

## Abstract

**Background:**

Key to a successful outcome of total knee arthroplasty (TKA) is to attain optimum alignment, adequate balance, and deformity correction. In primary TKA, this can be achieved efficiently by posterior stabilized (PS) design with or without the sub-periosteal release. However, certain circumstances such as post-traumatic arthritis are often associated with severe deformities with a significant bone defect, stiffness, and instability. Such deformities are extremely difficult to balance with soft tissue release only and require additionally constrained prostheses even in primary TKA. In such situation, constrained condylar knee (CCK) design is the ultimate choice. This study primarily aimed to report on clinical outcome, regain of function, and complication of patients who underwent primary CCK-TKA for severe deformity of the knee secondary to post-traumatic arthritis. The secondary aim was to find out the mid-term prostheses survival.

**Methods:**

Between February 2007 and November 2013, 38 consecutive patients with post-traumatic arthritis of the knee received cemented primary CCK-TKA. Thirty-four patients (21 men and 13 women) who had a minimum of 3 years follow-up were included in this retrospective study. We used Knee Society Score (KSS), Hospital for Special Surgery (HSS) score, and roentgenographic evaluation form to assess the patients. Prostheses survival was assessed using Kaplan-Meier’s survival analysis.

**Results:**

Patients were followed up for an average duration of 6.47 years. KSS knee score improved from 44 points (23–68) pre-operatively to 91 points (76–100) post-operatively [*P* < 0.001]. The average KSS functional score improved from 49 points (20–75) pre-operatively to 91 points (65–100) post-operatively [*P* < 0.001]. The average HSS score improved from 51 points (27–83) pre-operatively to 91 points (75–100) post-operatively [*P* < 0.001]. Similarly, the average ROM improved from 68.09° ± 35.99° (0**°**–120**°**) to 113.68° ± 8.90° (100**°**–130**°**) post-operatively [*P* < 0.001]. The average hip-knee-ankle (HKA) angle was 176.88° ± 14.48**°** (135**°**–199**°**) pre-operatively and 180.24° ± 1.77**°** (175**°**–184**°**) post-operatively. Radiolucencies were evident in 13 knees, mostly on the tibial side. Prostheses survival was 94.7% at a mean follow-up of 6.47 years.

**Conclusion:**

Despite severe deformity, instability, and stiffness at a relatively young age, mid-term follow-up of primary CCK-TKA in post-traumatic arthritis provides satisfactory clinical and functional outcomes with 94.7% prostheses survival. However, it is not without complication.

## Background

Intra-articular or peri-articular injuries are common in young and active individuals, which often lead to post-traumatic arthritis of the involved joint [1]. Prevalence of post-traumatic arthritis of the knee following injuries range from 21 to 44% [2] and is usually associated with severe bone loss and/or ligamentous injuries leading to a significant instability, stiffness, and severe deformity of the knee joint [3, 4].

Key to a successful outcome of total knee arthroplasty (TKA) is to attain optimum alignment, adequate balance, and deformity correction [5]. Generally, in primary TKA, this can be achieved efficiently by posterior stabilized (PS) design with or without the sub-periosteal release of ligaments. However, there are circumstances which require additionally constrained prostheses even in primary TKA such as severe bony defects and/or deformities and collateral ligament insufficiency that are extremely difficult to correct with soft tissue release only [5–7]. Soft tissue release in such situation somewhat jeopardizes the surrounding structures [8]. Additionally, in post-traumatic arthritis, there are always technical complexities owing to prior scars, the risk of infection, mal-alignment, knee stiffness, ligament insufficiency, and a significant bone loss. Such deformities warrant the use of constrained condylar knee (CCK) prosthesis with its variety of available stems and augments [3, 4, 9].

However, there are some disadvantages of CCK prostheses which include theoretical mechanical loosening due to load transfer to the respective bone ends via an intramedullary extension of the stems leading to early failure and a periprosthetic fracture [8, 10, 11]. Polyethylene insert wearing is another drawback of CCK [11]. Revision TKA following CCK prostheses is an extremely difficult procedure, as a need for stem removal increases significant morbidity and operating time [10].

Previous studies about the CCK-TKA focused mainly in two headings. First is the assessment of the effectiveness of CCK prostheses in different primary diagnoses including primary osteoarthritis, rheumatoid arthritis, post-traumatic arthritis, and Charcot arthropathy, leading to severe instability and/or deformity of the knee joint [7, 8, 10–13]. Second is the assessment of the outcome of primary TKA in post-traumatic arthritis using a variety of prostheses including cruciate retaining (CR), PS, CCK, or rotating hinge (RH) [3, 4, 14–16]. The majority of the studies reported improvement in the functional score post-operatively following CCK-TKA. However, till date, no study has focused on the use of CCK prostheses in primary TKAs following post-traumatic arthritis. This retrospective study primarily aimed to report on clinical outcome, regain of function, and complication of patients who underwent primary CCK-TKA for severe deformity of the knee secondary to post-traumatic arthritis with a minimum follow-up of 3 years. The secondary aim was to find out the mid-term prostheses survival.

## Methods

### Patients

Between February 2007 and November 2013, 38 consecutive patients with post-traumatic arthritis of the knee with severe deformity received cemented primary Zimmer® NexGen® Legacy® Constrained Condylar Knee (LCCK®) prostheses TKA. All the patients had unilateral CCK-TKA. Out of 38 patients, 1 patient died, 1 patient lost follow-up, 1 patient underwent implant removal secondary to prosthetic infection, and 1 patient had revision surgery for early prosthetic loosening, and they are excluded from the study though included in the prosthesis survival and complication rate. Eventually, 34 patients were available at the final follow-up and were included in the study. Detailed demographic characteristics of patients are well illustrated in Table 1. The average gap between trauma and CCK-TKA was 7.73 ± 6.37 years (1–26). There were 21 men and 13 women with an average age of 58 ± 9.88 years (33–73). Eighteen patients had left knee involvement, and 16 had right knee involvement. The average follow-up of patients was 6.47 ± 1.99 years (3–9.5). Out of 34 patients, 23 (67.6%) patients had knee range of motion (ROM) of 90° or less pre-operatively. Primary diagnosis included 20 (58.8%) proximal tibia fractures, 9 (26.5%) distal femur fractures, 4 (11.8%) both tibia and femur fractures, and 1 (2.9%) both tibia and femur with patella fractures. Thirty (88.3%) patients had a history of surgical management of the fracture, whereas 4 (11.7%) patients received conservative management of the fracture.

**Table 1:** Demographics and characteristics of patients [Mean ± SD, or *n* (%)]

Demographic parameters (*N* = 34)	Mean ± SD, or *n* (%)	Range
Age (years)	58 ± 9.88	33–73
Male/female	21/13	
Right/left	16/18	
BMI (kg/m^2^)	24.86 ± 1.27	21.80–27.6
Trauma to TKA gap (years)	7.73 ± 6.37	1–26
Prior surgery (yes/no)	30/4	
Follow-up (years)	6.47 ± 1.99	3–9.5
Primary diagnosis, *n* (%)	Proximal tibia fracture, 20 (58.8%)	
	Distal femur fracture, 9 (26.5%)	
	Tibia and femur fracture, 4 (11.8%)	
	Femur and patella fracture, 1 (2.9%)	

*N* total sample, *n* number of cases, *BMI* body mass index, *TKA* total knee arthroplasty, *SD* standard deviation

Inclusion criteria were as follows: (1) patients who underwent a primary CCK-TKA for post-traumatic arthritis during the given period and had at least 3 years follow-up and (2) patients with previous knee surgery, such as prior osteotomies around the knee and/or non-arthroplasty implants like osteosynthesis plates or intramedullary nails. Exclusion criteria were as follows: (1) CCK-TKA for reasons other than post-traumatic arthritis; (2) patients underwent prior TKA or conversion of a unicompartmental knee arthroplasty (UKA); (3) patients who did not have pre-operative or post-operative imaging; and (4) arthroplasty associated with oncologic resection.

### Surgical procedure

The decision to use CCK prosthesis was made by the experienced senior orthopedic surgeon (SHY) and his panel. Templating of pre-operative X-rays were performed on all knees. A standard medial parapatellar approach, anterior referencing, and measured resection technique were applied. Initially, tibial preparation was performed by a neutral tibial cut with 3**°** of the posterior slope with an extramedullary guide. An intramedullary guide with a broach was applied for measurement of appropriate distal femoral cut parallel to the trans-epicondylar axis in 5**°**–7**°** valgus angle of anatomical axis. The decision to use CCK prosthesis was made pre-operatively in 60% of patients on the basis of severe deformity and instability where soft tissue release was not attempted. In 40% of patients, the decision was made intra-operatively when there was no adequate balance in flexion and/or extension even after an adequate sub-periosteal soft tissue release. A spacer block was used to assess extension and flexion gaps. Appropriately sized tibial and femoral provisional with a proper thickness spacer block was inserted. The operating surgeon (SHY) then evaluated stability by applying varus-valgus stress in extension, mid flexion, and at 90**°** of flexion. The alignment was assessed using an extramedullary alignment rod. Finally, appropriately sized cemented tibial and femoral prostheses along with stems were implanted. Various bone graft or augments were used whenever there was a considerable bone defect. The lateral retinacular release was performed when intra-operative patella mal-tracking was evident. Routine patella resurfacing was not performed in our series; however, patella denervation with electrocautery was performed on all the knees.

### Rehabilitation

Antibiotics and analgesics were routinely administered and continued for 48 h post-operatively. All patients were administrated with prophylactic anticoagulants. Rehabilitation started the next day with passive mobilization, followed by active rehabilitation. Early weight-bearing was usually encouraged. Early and late complications such as venous thromboembolism (VTE), hemorrhage, infection, dislocation, loosening, and periprosthetic fractures were continuously monitored in the ward and during the follow-ups. Standing anteroposterior (AP) and lateral view X-rays of the operated knee were taken in 3 days post-operatively and every follow-up visit for evaluation.

### Clinical and radiological evaluation

The questionnaires were prepared according to the Knee Society Score (KSS) [17] and Hospital for Special Surgery (HSS) score [6]. KSS consisted of KSS knee score including ROM, and KSS functional score. Pre-operative data such as demographic information and knee scores were collected retrospectively through the hospital database. Post-operative data were collected during follow-up visits. Demographic information such as age, gender, body mass index (BMI), side of the operated knee (right/left), and primary diagnoses were recorded. Patients were requested via a telephone call to visit the hospital for final follow-up even if they had left the follow-up in between.

Radiographs were evaluated for mechanical alignment (hip-knee-ankle (HKA) angle), anatomic alignment, and any radiolucent lines using the Knee Society Roentgenographic Evaluation form [18]. The last follow-up X-rays were compared with the immediate post-operative X-rays to look for the position of the implant, aseptic loosening, periprosthetic fracture, heterotrophic ossification (HO), and any other complications. The clinical and radiological evaluations were carried out by the orthopedic surgeon (XZL) who was not involved in the operation.

### Statistical analysis

We used Statistical Package for Social Sciences (IBM SPSS Statistics 23) version 23 for statistical analysis. Categorical data were analyzed using *chi*-*square* test, and *paired t* test was chosen for analysis of continuous parametric data, whereas the *Mann-Whitney U* test was used to analyze nonparametric continuous data. *Kaplan-Meier’s* survival analysis was used for prostheses survival. Results of categorical data were presented as frequencies and percentages, whereas results of continuous data were presented as the mean ± standard deviation (SD) and range. Statistical differences were considered significant for *P* values < 0.05.

## Results

### Clinical results

Clinical scores are well illustrated in Table 2. The average KSS knee score improved from 44 points (23–68) pre-operatively to 91 points (76–100) at the final follow-up [*P* < 0.001]. Out of 34 patients, 32 (94.1%) patients were rated as excellent and 2 (5.9%) patients were rated as good. The average KSS functional score improved from 49 points (20–75) pre-operatively to 91 points (65–100) at the final follow-up [*P* < 0.001]. The average HSS score improved from 51 points (27–83) pre-operatively to 91 points (75–100) at the final follow-up [*P* < 0.001].

**Table 2:** Pre-operative versus post-operative outcomes of primary CCK-TKA in post-traumatic arthritis where all the parameters significantly improved post-operatively

Parameters	Pre-operative	Post-operative	*P* value
(*N* = 34)	Mean ± SD (Range)	Mean ± SD (Range)	
KSS Knee Score	44.88 ± 13.27 (23–68)	91.82 ± 6.25 (76–100)	< 0.001*
KSS Functional Score	49.12 ± 14.16 (20–75)	91.03 ± 9.75 (65–100)	< 0.001*
Hospital for Special Surgery (HSS) score	51.00 ± 13.49 (27–83)	91.68 ± 7.20 (75–100)	< 0.001*

*Statistically significant differences were observed in all the parameters

*N* sample size, *SD* standard deviation, *KSS* Knee Society Score

**Fig. 1:**
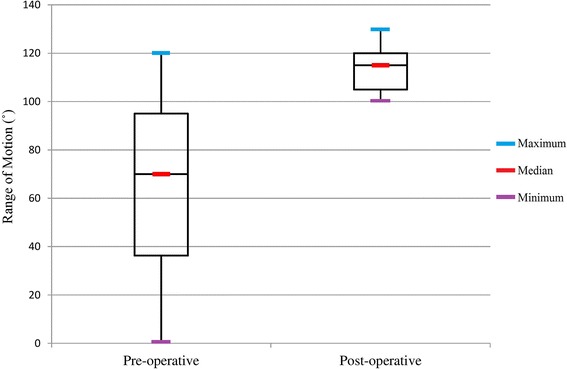
The pre-operative and post-operative range of motion (ROM) of patients

Similarly, the average ROM improved from 68.09° ± 35.99° (0**°**–120**°**) to 113.68° ± 8.90° (100**°**–130**°**) at the final follow-up [*P* < 0.001] (Fig. 1). Pre-operatively, 23 (67.6%) patients had average flexion contracture of 17° (5**°**–45**°**); post-operatively, 1 (2.9%) patient had flexion contracture of 5°. Similarly, 2 (5.9%) patients had pre-operative extension lag of 5°, but none of the patients had extension lag post-operatively.

### Radiographic results

**Fig. 2:**
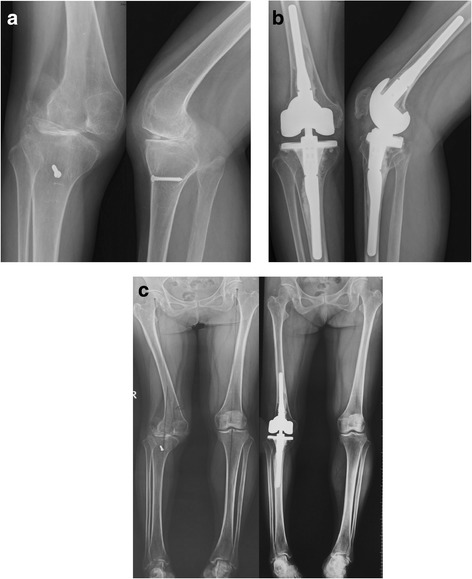
Implantation of CCK prosthesis in a 62-year-old female with post-traumatic arthritis with valgus deformity and significant instability of the right knee. **a** Pre-operative anteroposterior (AP) and lateral radiographs; she had undergone some type of procedure on her index knee. **b** Post-operative radiograph shows 3° of valgus and no radiolucent lines. **c** Comparison of pre-operative and post-operative long leg radiographs

**Fig. 3:**
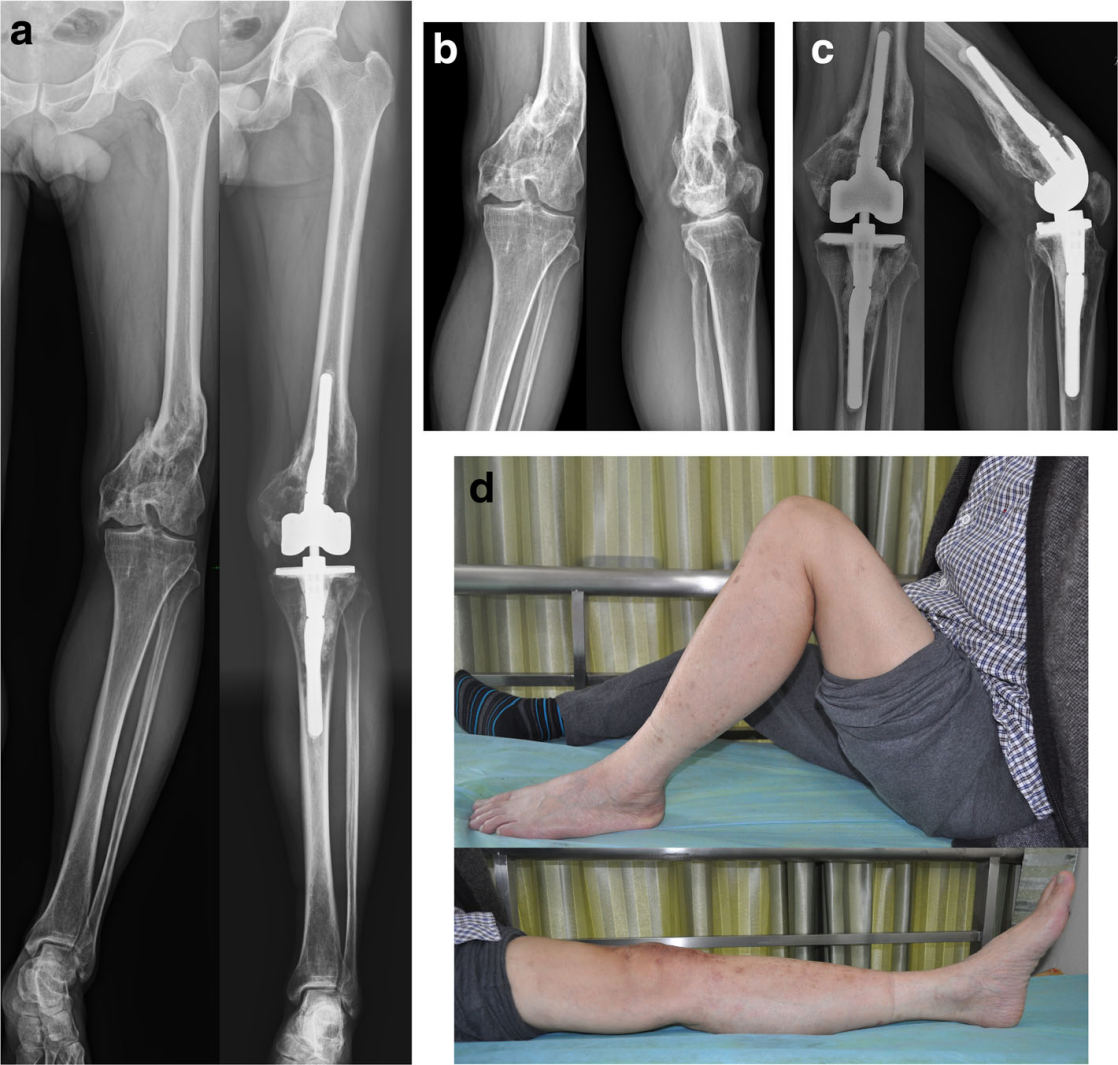
CCK prosthesis in a 58-year-old male patient with the mal-united distal femur and post-traumatic arthritis of the left knee. The patient was having knee stiffness and patella baja for 20 years due to post-traumatic scar. **a** Comparison of pre-operative and post-operative long leg radiographs in anteroposterior (AP) view. **b**, **c** Pre-operative and post-operative radiographs respectively. **d** Clinical photographs showing a post-operative range of motion (ROM)

The pre-operative radiographic evaluation showed an average varus deformity of 12° (0**°**–30°) in 10 (29.4%) patients, and an average valgus deformity of 15° (7.5-25) in 17 (50%) patients in weight-bearing AP radiographs, whereas 7 (20.6%) patients had a normal valgus angle of 3° to 7°. There were no deformities at the final follow-up. The average HKA angle was 176.88° ± 14.48**°** (135**°**–199**°**) pre-operatively and 180.24° ± 1.77**°** (175**°**–184**°**) at the final follow-up (Figs. 2 and 3). Radiolucencies were evident in 13 knees, 10 were on the tibial side and 3 on both sides. However, all these radiolucencies were less than 1 mm in size and were evident also in immediate post-operative radiographs.

### Prosthesis survival

We used endpoint as a revision for any reason for all 38 cases. Prostheses survival was 94.7% (95% confidence interval (CI) 82.3~ 99.4%) at an average follow-up of 6.47 years.

### Complication

Complications are well illustrated in Table 3. Two patients, a patient with an implant loosening in 1 month and a patient with prosthetic infection in 2.5 years post-operatively, underwent prostheses removal followed by revision procedures. A patient who suffered non-displaced periprosthetic fracture around the tibial stem which was managed conservatively reported a good outcome. Three patients had symptomatic deep vein thrombosis (DVT); however, all of them recovered well after early diagnosis and essential management. Common peroneal nerve palsy occurred in one patient, whose motor symptoms recovered after 6 months post-operatively. Heterotropic ossification (HO) occurred in one, and asymptomatic patella baja occurred in one patient.

**Table 3:** Complications of primary CCK-TKA in post-traumatic arthritis (38 knees)

Complications	*Knees, n* (%)	Revision *n* (%)
Deep venous thrombosis	3 (7.89)	
Infection	1 (2.63)	1 (2.63)
Periprosthetic fracture	1 (2.63)	
Prosthetic loosening	1 (2.63)	1 (2.63)
Common peroneal nerve injury	1 (2.63)	
Heterotropic ossification	1 (2.63)	
Patella baja	1 (2.63)	
Overall	9 (23.68)	2 (5.26)

*n* number of cases

## Discussion

The most important finding of our study was that the primary CCK-TKA successfully corrected the severe deformity with significant bone defects, stiffness, and instability of the knee joint secondary to post-traumatic arthritis with prostheses survival rate of 94.7% restoring excellent clinical outcome and regain of function. However, post-operative complication rate was as high as 24%.

Previously, Lachiewicz and Soileau [13] studied the results of second-generation primary CCK-TKA in 27 knees; there was a significant improvement in all the parameters including KSS knee score, KSS functional score, and HSS score post-operatively at the final follow-up. Using KSS knee score, 12 (44%) knees rated as excellent, 14 (52%) as good, and 1 (4%) as fair. Ye et al. [19] compared the outcome of CCK-TKA in complex primary and revision surgery and reported no significant difference between two groups at a mean follow-up of 5.5 years post-operatively. However, they found a significant improvement in the clinical scores with 92% knees as having good to excellent results in the primary CCK group [19]. Moreover, Lizaur-Utrilla et al. [20] provided a similar opinion with a significant improvement post-operatively yet found no significant differences in the post-operative outcomes of the patients who underwent primary TKA for post-traumatic arthritis versus primary osteoarthritis. However, Lunebourg et al. [15] reported that regardless of having a significant difference in the post-operative scores of primary TKA, the post-operative scores of TKA following post-traumatic arthritis was lower than that of the primary osteoarthritis group. This difference was possibly due to the poor pre-operative status of patients, not because of the intrinsic success of the procedure [15]. In our study, mid-term results of cemented primary CCK-TKA in post-traumatic arthritis was evaluated using KSS knee score, KSS functional score, and HSS score. Regardless of having severe deformity, stiffness, and instability pre-operatively, 94% patients had excellent post-operative scores at the last follow-up, which was comparable with reports mentioned above.

Another important finding of our study was a significant improvement in the post-operative ROM. Although our patients had poor preoperative ROM with an average of 68°, it improved significantly with an average ROM of 113° at the final follow-up. Our finding was consistent with previous reports as the majority of studies reported post-operative ROM of CCK prostheses ranging from 89.4**°** to 117° [8, 11, 21–24]. Maynard et al. [24] reported post-operative ROM to be dependent on pre-operative ROM. In our study, even though 23 (67.6%) knees had a pre-operative ROM of 90° or less, post-operatively the ROM improved satisfactorily with all having ROM of 100° or more. Considering this report, we can conclude that post-operative ROM depends not only on pre-operative ROM, but also on adequate intra-operative balancing, and appropriate rehabilitation.

Despite having a significant improvement in clinical and functional outcome following TKA in post-traumatic arthritis, the complication rate is often high (Table 4) [2]. Complications include infection, wound complications, intra-operative rupture of the patellar tendon, stiffness, and mechanical loosening [2]. Various factors contribute to the complications after TKA in post-traumatic arthritis such as prior fracture surgeries, scarring of soft tissue, severe joint deformity, and mal-positioning of the implants. Piedade et al. [25] reported that previous surgery around the knee predisposes to higher-rate post-operative complications after TKA. Prosthetic infection is regarded as most frequent and a dreadful complication following TKA and is the primary reason for re-operation [11]. Jamsen et al. [26] reported that prior fracture or use of constrained prosthesis was a risk factor for infection. In our study, overall complications occurred in 9 (23.7%) knees out of 38 patients as mentioned above and 2 (5.26%) patients (2 knees) needed revision TKA. Previous studies reported that the overall infection rate in primary TKA secondary to post-traumatic arthritis was between 3.4 and 9.6% [3, 15, 16, 20, 27–29]. Regardless of the use of CCK in a relatively vulnerable group of patients, prosthetic infection occurred in only one (2.63%) patient in 2.5 years following primary TKA that needed prosthetic removal. A relatively low infection rate might be due to strict intra-operative aseptic precautions along with the routine use of prophylactic antibiotics. Another revision occurred in a male patient with distal femoral fracture with prior surgeries followed by knee stiffness and considerable deformity for more than 20 years. Primarily, we used LCCK prosthesis with stem extension in femoral component and without stem extension in the tibial component; unfortunately, aseptic tibial component prosthetic loosening occurred at 1 month following TKA necessitating a revision surgery with a long stem. We regarded the reason for loosening to be wrong prosthesis choice leading to mal-positioning [3, 16]. In this study, we found the survival rate prostheses to be 94.7% (95% CI 82.3~99.4%) and was fairly consistent with the previous literature of long-term follow-up when the revision of any cause was set as an endpoint [11, 21].

**Table 4:** A literature review of complications rate of primary TKA after post-traumatic arthritis

	References							
Parameters	Massin et al. [30]	Abdel et al. [27]	Lonner et al. [29]	Papadopoulos et al. [28]	Parratte et al. [16]	Lunebourg et al. [15]	Lizaur-utrilla et al. [20]	Weiss et al. [3]
Sample size (*N*)	40	62	31	48	74	33	29	110
Male/female	21/19	22/40	15/15(1-BL)	10/37(1-BL)	34/40	18/15	10/19	32/77(1-BL)
Age (years)	59	63	60	65	63	69	57	64
Follow-up (years)	5	15	4	6	4	11	7	5.5
Complications *n* (%)								
Venous thrombosis		1(1.6)	2(6.4)					6(5.5)
Aseptic failure			8(25.8)		1(1.7)	1(3.0)		4(3.6)
Infection	2(5.0)	5(8.0)	3(9.6)	3(6.2)	4(6.8)	2(6.1)	1(3.4)	5(4.5)
Stiffness		6(9.6)			6(10.2)	2(6.1)	1(3.4)	8(7.3)
Patellar tendon rupture	3(7.5)		1(3.2)	1(2.1)	3(5.1)		1(3.4)	
Ligament rupture		1(1.6)						1(0.9)
Patella subluxation		2(3.2)	2(6.4)	1(2.1)				
Patella clunk syndrome					1(1.7)	1(3.0)		1(0.9)
Skin necrosis and wound dehiscence	2(5)	3(4.8)	2(6.4)	2(4.2)			1(3.4)	5(4.5)
Peroneal nerve injury					1(1.7)			
Popliteal artery injury	1(2.5)				1(1.7)			
Periprosthetic fractures	1(2.5)	1(1.6)						
Pseudoarthrosis	1(2.5)							
Instability					1(1.7)			2(1.8)
Heterotropic ossification						1(3.0)		
Hematoma		1(1.6)		2(4.2)	2(3.4)			
Reflex sympathetic dystrophy		1(1.6)						1(0.9)
Overall	11(27.5)	21(33.8)	18(58)	9(18.7)	20(33.9)	7(21.2)	4(13.7)	33(30.0)

*N* sample size, *n* number of cases, *BL* bilateral

Our study has several limitations. Our study has all the biases related to a retrospective cohort study with a relatively small sample size. Selection bias is another limitation as the choice of the prosthesis was at the decision of the chief operating surgeon. Lastly, the results presented here are from a single hospital and may reflect regional and institutional bias.

## Conclusion

This study demonstrates that despite having a severe deformity and stiffness of the knee joint at a relatively young age, mid-term follow-up of primary CCK-TKA in post-traumatic arthritis provides satisfactory clinical and functional outcomes with 94.7% prostheses survival. Considering this report, we can conclude that CCK-TKA is effective and a good option for the treatment of post-traumatic arthritis of the knee. However, it is not without complication. Long-term follow-up is warranted to obtain an ultimate conclusion.

## Abbreviations

AP: Anteroposterior
BMI: Body mass index
CCK: Constrained condylar knee
CR: Cruciate retaining
HKA: Hip-knee-ankle
HO: Heterotrophic ossification
HSS: Hospital for Special Surgery score
KSS: Knee Society Score
PS: Posterior stabilized
RH: Rotating hinge
ROM: Range of motion
TKA: Total knee arthroplasty
UKA: Unicompartmental arthroplasty
VTE: Venous thromboembolism
